# Differences in Microbial Community Composition between Uterine Horns Ipsilateral and Contralateral to the Corpus Luteum in Beef Cows on Day 15 of the Estrous Cycle

**DOI:** 10.3390/microorganisms11082117

**Published:** 2023-08-20

**Authors:** Madison Blake Walker, Matthew Patrick Holton, Todd Riley Callaway, Jeferson Menezes Lourenco, Pedro Levy Piza Fontes

**Affiliations:** Department of Animal and Dairy Science, University of Georgia, Athens, GA 30602, USA; madison.walker@uga.edu (M.B.W.); mpholton@uga.edu (M.P.H.); todd.callaway@uga.edu (T.R.C.); jefao@uga.edu (J.M.L.)

**Keywords:** bovine, estrus, reproduction, uterine environment, uterine microbiome

## Abstract

This study evaluated differences in uterine microbiota composition between uterine horns ipsilateral and contralateral to the corpus luteum of beef cows on day 15 of the estrous cycle. Cows (*n* = 23) were exposed to an estrus synchronization protocol to exogenously induce synchronized ovulation. Cows were then euthanized on day 15 of the estrous cycle, and individual swabs were collected from uterine horns ipsilateral and contralateral to the corpus luteum using aseptic techniques. DNA was extracted, and the entire (V1–V9 hypervariable regions) 16s rRNA gene was sequenced. Sequences were analyzed, and amplicon sequence variants (ASVs) were determined. Across all samples, 2 bacterial domains, 24 phyla, and 265 genera were identified. *Butyribirio*, *Cutibacterium*, *BD7-11*,* Bacteroidales BS11 gut group*,* Ruminococcus*,* Bacteroidales RF16 group*,** and *Clostridia UCG-014* differed in relative abundances between uterine horns. *Rikenellaceae RC9 gut group*,* Bacteroidales UCG-001*,* Lachnospiraceae AC2044 group*,* Burkholderia-Caballeronia-Paraburkholderia*,* Psudobutyribibrio*,** and an unidentified genus of the family *Chitinophagaceae* and *dgA-11 gut group* differed between cows that expressed estrus and those that did not. The composition of the microbial community differed between the ipsilateral and contralateral horns and between cows that expressed estrus and cows that failed to express estrus, indicating that the uterine microbiota might play a role in cow fertility.

## 1. Introduction

Embryonic mortality is a major contributor to infertility and subfertility in all mammalian species, including humans [1]. In cattle, reproductive failure costs beef and dairy producers more than USD 1 billion annually [2]. In beef production systems, fertilization rates exceed 80% of females exposed to artificial insemination; however, only approximately 50% of females are able to successfully establish pregnancy [3], indicating that pregnancy loss during early embryonic development is a major contributor to reproductive failure. The majority of these losses occur between days 6 and 20 of gestation [4,5], a pivotal period of pregnancy during which the bovine conceptus undergoes dramatic morphological and functional changes prior to implantation [6,7,8]. Collectively, these changes result in an orchestrated paracrine communication between the elongating conceptus and the endometrium that is required for successful pregnancy establishment [7,9]. Disruptions to the uterine environment that impede the adequate growth and development of the conceptus lead to early embryonic mortality [9].

Changes in endometrial transcriptome and histotrophic composition that occur during early pregnancy are predominantly regulated by circulating progesterone produced by the corpus luteum (CL) during diestrus [10]. The endometrial concentration of progesterone is greater in the uterine horn ipsilateral to the CL compared with the contralateral horn [11,12]. There are also differences in endometrial transcriptome between ipsilateral and contralateral uterine horns during diestrus [13], indicating not only a local effect of the CL on endometrial progesterone concentrations but also an effect on endometrial function. Interestingly, pregnancy establishment is decreased when embryos are transferred in the contralateral horn compared with transfers performed in the ipsilateral horn [14], further highlighting the unequivocal role of progesterone modulating local uterine function and pregnancy establishment.

Cows that display estrus have increased plasma concentrations of estradiol prior to ovulation and increased pregnancy rates in both artificial insemination [15,16] and embryo transfer settings [17]. Cows that express estrus also have decreased pregnancy loss after a pregnancy is initially confirmed via ultrasonography during early gestation [16,17]. Hence, the greater fertility observed in cows that express estrus is not explained only by improved follicular and oocyte development [18,19] but also changes in subsequent luteal development and the resulting uterine environment [20]. Next-generation sequencing technologies have allowed us to discover the presence and composition of a microbiome in the bovine uterus; however, the reason for its existence or role in reproduction remains poorly understood [21,22,23]. We hypothesized that there were differences in the microbial community of the ipsilateral and contralateral uterine horns on day 15 of the estrous cycle in *Bos taurus* beef cows. Moreover, we hypothesized that there are differences in the uterine microbial community composition between cows that display estrus and those that do not express estrus. Therefore, the objectives of this study were to: (1) evaluate the differences in the uterine microbial community between the ipsilateral and contralateral uterine horns on day 15 of the estrous cycle, and (2) evaluate the impact of estrus expression on subsequent uterine microbial composition.

## 2. Material and Methods

### 2.1. Animals and Experimental Design

All procedures were carried out in accordance with the recommendations of the Institutional Animal Care and Use Committee at the University of Georgia, Athens (Protocol A2023 03-024-Y1-A0). Non-pregnant Angus (*Bos taurus*) cows (*n* = 23; body weight = 582 ± 68.6 kg; body condition score = 5.46 ± 0.67; age = 5.9 ± 3.1) from the University of Georgia’s Northwest Georgia Research and Education Center (Rome, GA 34.34° N, 85.12° W) were utilized in this experiment. Only cows that were free of physical abnormalities and had no uterine or ovarian abnormalities following a gynecological ultrasound examination were utilized in the present study. All cows were born in the same operation and exposed to the same environmental conditions. Cows were housed as a single group on native improved bermudagrass (*Cynodon dactylon*) pasture and had ad libitum access to bermudagrass hay and mineral supplementation. Before the initiation of the study, cows were exposed to a modified estrus synchronization program to induce synchronized ovulation without the use of intravaginal progesterone inserts. Briefly, cows received a 25 mg injection of prostaglandin F_2α_ (PG; 2 mL Lutalyse HighCon, Zoetis Animal Health, Parsippany-Troy Hills, NJ, USA) on day −13, followed by a 100 μg injection of gonadotrophin-releasing hormone (GnRH; 2 mL Factrel; Zoetis Animal Health) on day −10. Another 25 mg injection of PG was administered on day −3 to induce luteolysis, and an estrus detection patch (Estrotect Breeding Indicator, Rockway Inc., Spring Valley, WI, USA) was applied. Cows received a second GnRH injection on day 0, and estrus detection patches were evaluated. Cows were considered to have expressed estrus when at least 50% of the rub-off coating was removed from the estrus detection patch. B-mode ultrasonography (Easi-Scan:Go; IMV Imaging, Rochester, MN, USA) was used to confirm synchrony through ovarian mapping on days −13, −10, −3, 0, and 14. Cows were considered to have ovulated to the first GnRH injection (d −10) when a CL was present on day −3 on the same ovary that a dominant follicle (>8 mm) was present on day −10. Cows were considered to have ovulated spontaneously or in response to the second GnRH (day 0) when CL was present on day 14 on the same ovary that a dominant follicle (>8 mm) was present on day 0. Only cows that responded to both GnRH injections without double ovulation were used for sample collection. All cows were transported to a commercial packing plant (FPL Foods, Augusta, GA (33.46° N, 81.96° W)) on day 14 and harvested on day 15 of the study.

### 2.2. Uterine Microbiome Sample Collection

Cows were harvested, and the reproductive tracts (including vagina, cervix, uterus, and ovaries) were removed. Each uterus was washed with water, and then the incision site was sterilized with 70% ethanol. An aseptic cross-sectional incision was made into each horn at the greater curvature region and a sterile cotton tipped swab inserted into the cranial portion of the uterine horn, rubbed against the uterine epithelial lining until saturated, and immediately flash frozen (−80 °C) using liquid N_2_ [24].

### 2.3. DNA Extraction and Sequencing

Samples were thawed to room temperature, and 1 mL of sterile phosphate-buffered saline (PBS) was added to each tube. Tubes were vortexed for 10 min to maximize recovery. DNA extraction was performed using a commercial kit (QIAamp BiOstic Bacteremia DNA kit; QIAGEN, Venlo, The Netherlands) according to manufacturer instructions using 0.5 mL of each sample. The concentration of DNA was quantified via spectrophotometry (Synergy H4; BioTek, Winooski, VT, USA). The entire 16S rRNA gene libraries were prepared from genomic DNA using LoopSeq kits (Loop Genomics, San Jose, CA, USA), and synthetic long reads were constructed from the short-read sequences generated through Illumina sequencing technology [25]. Analysis of sequences was performed using the Quantitative Insights Into Microbial Ecology (QIIME) bioinformatics pipeline, version 2-2021.11 [26]. Sequences were cleaned and assigned to taxa using a pre-trained naïve Bayes classifier [27,28], which was trained on the full-length small subunit of the SILVA 138 database [29]. Samples were rarified to a common depth of 154 sequences for computation of alpha and beta diversity metrices and to calculate the mean relative abundance of individual taxa. One cow was removed from the study due to receiving antibiotic treatment during the synchronization protocol. Hence, sequence analysis was performed on samples from 22 cows (Table 1). After all quality filtering steps, a total of 36 samples (17 contralateral and 19 ipsilateral) were analyzed. After filtering and rarefaction, 15 cows retained both ipsilateral and contralateral samples. Moreover, there were 15 samples from cows that did not display estrus and 21 from cows that did display estrus, including matched pairs of 8 cows that displayed estrus and 7 that did not.

### 2.4. Statistical Analysis

Data were analyzed using a python script [30] to preform Kruskal–Wallis tests for all the independent variables. In addition, for cows that had high-quality microbial data from both horns, paired *t*-tests and Wilcoxon signed-rank tests were performed for the explanatory variables. The computed alpha diversity indexes were as follows: number of observed features (ASVs), Faith’s phylogenetic diversity, Shannon index, and Pielou’s evenness. To incorporate phylogenetic relatedness, beta diversity was calculated using the unweighted UniFrac distance matrix [31], and differences between groups were determined via permutational multivariate analysis of variance (PERMANOVA). For all statistical tests, significance was declared when *p* ≤ 0.05, and tendencies when 0.05 < *p* ≤ 0.10. When applicable, the standard error of the mean (SEM) was shown as a measure of dispersion of the data.

## 3. Results

**Figure 1:**
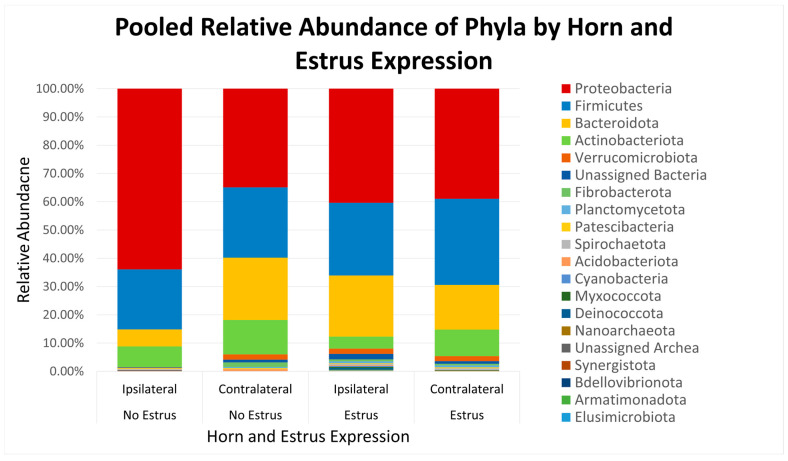
Relative abundance of phyla based on estrus expression and uterine horn position relative to the luteal bearing ovary (ipsilateral or contralateral) in non-pregnant beef cows on day 15 of the estrous cycle. Estrus: expressed estrus prior to ovulation. No estrus: failed to express estrus prior to exogenously induced ovulation.

**Figure 2:**
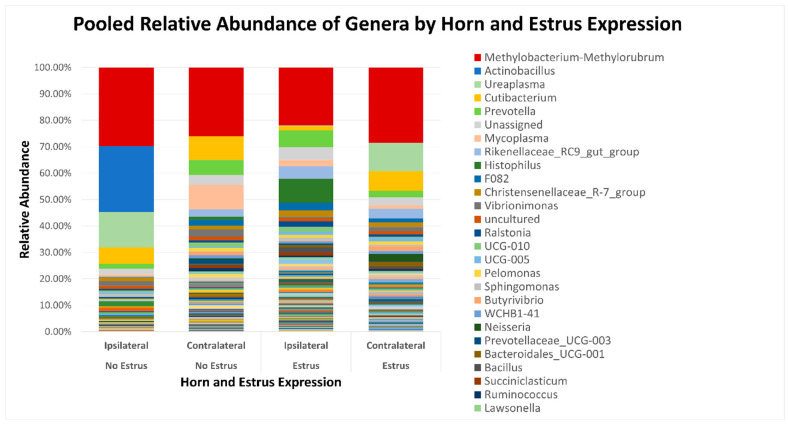
Relative abundance of genera based on estrus expression and uterine horn position relative to the luteal bearing ovary (ipsilateral or contralateral) in non-pregnant beef cows on day 15 of the estrous cycle. Estrus was evaluated on experimental day 0 using an estrus detection aid and cows were considered to have expressed estrus when ≥50% of the rub-off coating was removed from the estrus detection patch.

**Figure 3:**
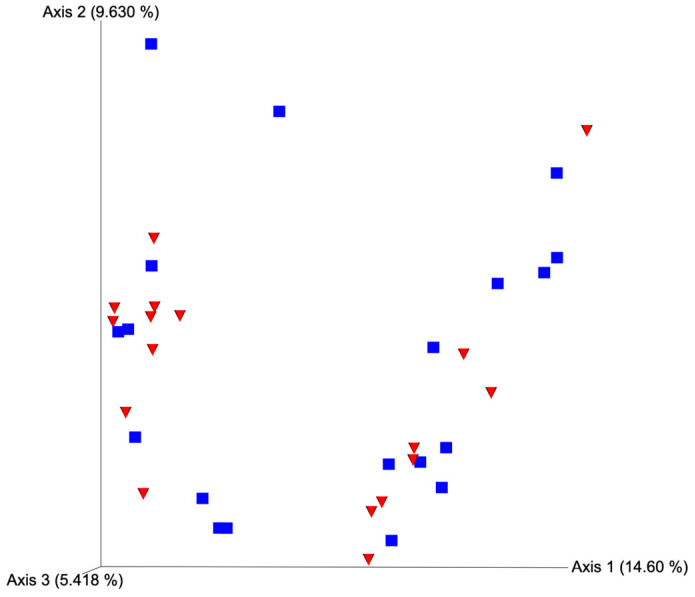
Principal coordinate analysis plot for unweighted UniFrac distances based on horn position relative to the luteal bearing ovary (ipsilateral or contralateral) in non-pregnant beef cows on day 15 of the estrous cycle. Blue squares and red triangles represent ipsilateral and contralateral uterine horns, respectively. PERMANOVA *p*-value = 0.378.

**Figure 4:**
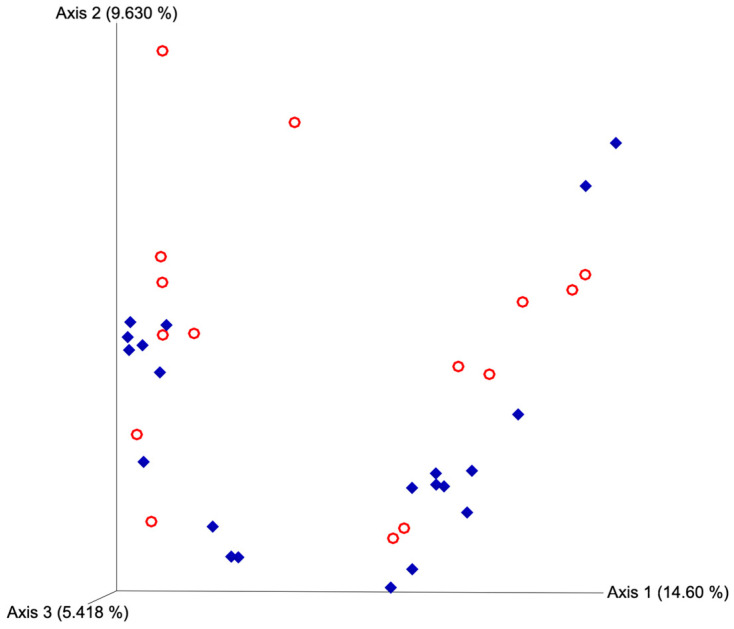
Principal coordinate analyses plot for unweighted UniFrac distances based on estrus expression. Blue diamonds represent cows that expressed estrus and red circles represent cows that failed to express estrus. Estrus was evaluated on experimental day 0 using an estrus detection aid and cows were considered to have expressed estrus when ≥50% of the rub off coating was removed from the estrus detection patch. PERMANOVA *p*-value = 0.146.

After quality filtering and taxonomic classification, a total of 2 kingdoms, 24 phyla, and 265 genera were assigned to sequences. The relative abundances of all phyla did not differ between horns or between estrus expression (Figure 1). At the phylum level, samples from the ipsilateral horn of cows that did not express estrus had a greater (~23%) relative abundance of Proteobacteria but lower relative abundances of Bacteroidota (Figure 2). In addition, there were no differences (*p* ≥ 0.334) in alpha diversity metrics between uterine horns (Table 1). The data approached a tendency for the increased (*p* = 0.131) alpha diversity based on the number of observed features and tended to have increased (*p* = 0.098) alpha diversity based on the Shannon index of cows that expressed estrus when compared with cows that failed to express estrus (Table 2). There was also no clear clustering in the principal coordinate analysis of unweighted UniFrac distances based upon uterine horn (Figure 3). Similarly, the principal coordinate analysis for unweighted UniFrac distances showed no clear clustering of the ipsilateral samples for cows that displayed estrus and those that did not (Figure 4).

At the genus level, *Butyribirio*,* Cutibacterium*,* BD7-11, Bacteroidales BS11 gut group*, and *Ruminococcus* had greater (*p* ≤ 0.045) relative abundances in the contralateral horn compared to the ipsilateral horn (Table 3). However, *Bacteroidales RF16 group* and *Clostridia UCG-014* were more abundant (*p* ≤ 0.025) in the ipsilateral than the contralateral horn (Table 3). Paired *t*-test comparisons revealed that uterine horns ipsilateral to the CL had greater abundance (*p* ≤ 0.045) of *Butyribirio*,* Cutibacterium*,* Ruminococcus*,* Bacillus*, and *Bacteroidales BS11 gut group* compared with the contralateral horns (Table 4). In addition, cows that expressed estrus signs had increased (*p* ≤ 0.045) abundances of Rikenellaceae RC9 gut group, Bacteroidales UCG-001, Lachnospiraceae AC2044 group, Burkhold-eria-Caballeronia-Paraburkholderia, and Pseudobutyribibrio than cows who did not express estrus (Table 5). Estrus expression resulted in decreased (*p* ≤ 0.035) relative abundances of an unidentified genus of the family Chitinophagaceae, Vibrionimonas, and dgA-11 gut group when compared to cows that did not display estrus (Table 5).

## 4. Discussion

The healthy uterus was long thought to be a sterile environment. However, recent developments in sequencing technology allowed for the characterization of the bovine uterine microbial community [22,23,32]. Most uterine microbiome studies in the bovine utilized short-sequence technology and only amplified a portion of the 16s rRNA gene [32,33,34]. In the present study, all nine hypervariable regions of the 16s rRNA gene were sequenced to increase the specificity and accuracy of taxonomic assignment [35]. To our knowledge, this is also the first bovine uterine microbiome study that utilized a surgical collection procedure to ensure a uterine sample free of fecal and vaginal microbial contamination. The large number of samples with few, or no, sequences recovered, even after polymerase chain reaction amplification, confirms that this approach produced low-contamination samples. Moreover, the use of whole 16s rRNA gene sequencing allowed for more taxa to be assigned to the genus and species levels (e.g., *Actinobacillus seminis* and *Brevibacterium casei*). This granularity of data allowed for observational differences that were not evident at higher levels of analyses (such as phylum or family), which would have not been identified with short sequencing due to a high number of unassigned taxa [36].

Alpha and beta diversity metrics have gained popularity as a convenient way to compare similarities or differences between two or more microbial communities [37]. Alpha diversity quantifies the amount of diversity within a particular community, whereas beta diversity quantifies species composition differences between two communities [38]. Though we lacked the statistical power to detect differences in alpha diversity in these conditions, it is a common assumption that greater diversity is most often associated with “healthy” microbiomes, and a loss in this biodiversity could be indicative of a disease state [39,40,41,42]. The tendency for decreased observed features and Shannon index in the ipsilateral horn of cows that did not express estrus may indicate a suboptimal uterine microbiome state for conceptus development and be associated with fertility differences seen between cows expressing estrus versus those that do not [15,17]. There were no beta diversity differences in the present study, which implies that the overall structure of the bacterial communities found across both horns and categories of estrus expression was similar and that these communities only differed in the relative abundance of specific taxa.

There was a tendency for an increase in populations of *Proteobacteria* and an accompanying tendency for decreased *Bacteriodota* in the ipsilateral horns of non-estrual compared with estrual cows. This is particularly intriguing since many members of *Proteobacteria* can cause disease (opportunistic), whereas most *Bacteriodota* are considered commensal [43,44]. Potentially, differences in the uterine environment between uterine horns are associated with dysbiosis in the ipsilateral horn of non-estrual cows, which contributes to decreased fertility compared to their estrual counterparts [15,17]. *Actinobacillus seminis* was detected exclusively in the horn ipsilateral to the CL in two cows that did not display estrus. *Actinobacillus seminis* is an opportunistic pathogen that has been shown to cause epididymitis and orchitis in rams, as well as metritis and abortion in ewes [45,46]. This bacterium has not been previously found in cattle, so its effect on fertility in this species is still unclear.

*Chitinophage* is a family that was present in nearly every sample and found across uterine horns and estrus expression. Chitin is a carbohydrate that characterizes fungi that some species in the family *Chitinophage* are able to use as a substrate for metabolism [47]. This result could indicate that there is some interaction between fungi present in the uterus and this bacterial population. The presence of fungal species, such as *Aspergillus fumigatus*, *Penicillium* spp., and *Candida kefyr*, in the uterus of cows has been reported and associated with fungal endometritis [48,49]. A decreased abundance of *Chitinophage* in cows that displayed estrus could indicate a lower population of fungi, further supporting the idea that the cows that did not display estrus have a uterine dysbiosis that could be impacting fertility. It must be noted that the present study did not attempt to quantify the presence of fungi in these samples.

The underlying reasons why the ipsilateral and contralateral microbial communities differed remain to be fully understood and are almost certainly multifaceted [11,13,20]. Even though transcriptome studies show large differences between the endometrial transcriptome of uterine horns during early compared with late diestrus when a conceptus is present [50], we were still able to detect differences in the microbial population during late diestrus. In addition, the presence of a conceptus influences the gene expression in the uterine transcriptome [13,50]. Thus, the presence of a conceptus may also impact the composition of the microbial community in the uterine horn, as local substrate availability and concentrations of various hormones (e.g., progesterone and estradiol) could alter the microbial population differently within each uterine horn during the late luteal phase. Progesterone concentrations vary throughout different parts of the estrous cycle, reaching their peak between days 8 and 18 [51]. Progesterone has an inhibitory effect on the innate inflammatory immune response [52,53], which could provide an opportunity for microbes to colonize and proliferate in the uterus during this time.

The results of this study support previous research in concluding that the healthy non-pregnant bovine uterus is not sterile. Our results also indicate that the uterine microbiome varies among different locations within the uterine lumen and might contribute to locally modulating the uterine environment in the uterine horn ipsilateral to the CL to favor conceptus development. Moreover, the results reported herein indicate that future studies investigating the role of the uterine microbiome on endometrial function and pregnancy establishment should be designed to account for variations in the uterine microbiome among different parts of the uterus. Differences in the uterine microbiome presented herein between cows that expressed estrus and cows that failed to express estrus highlight the impact of estrus expression on uterine biology during the subsequent diestrus. Further research is required to better understand the role of these differences in uterine microbiome on pregnancy establishment and, consequently, fertility in the bovine.

## Figures and Tables

**Table 1:** Differences in alpha diversity between ipsilateral and contralateral uterine horns on day 15 of the estrous cycle of non-pregnant beef cows ^1^.

Diversity Metric	Ipsilateral	Contralateral	*SEM*	*p*-Value (Paired *t*-Test)	*p*-Value (Wilcoxon)
Faith’s Phylogenetic Diversity	8.78	9.56	0.42	0.176	0.334
Observed Features (ASVs)	36.21	37.65	3.04	0.403	0.962
Shannon Index	3.65	4.04	0.25	0.188	0.825
Pielou’s Evenness	0.71	0.79	0.03	0.071	0.506

^1^ Uterine microbiome samples were collected on day 15 of the estrous cycle from the uterine horns ipsilateral and contralateral to the corpus luteum for entire (V1–V9 hypervariable regions) 16s rRNA gene sequencing. Non-parametric test (Wilcoxon) is also shown because some data failed to meet the assumptions of normality. SEM: Standard error of the mean.

**Table 2:** Impact of estrus expression on ipsilateral uterine horn alpha diversity metrics on day 15 of the estrous cycle in non-pregnant beef cows ^1^.

Diversity Metric	Estrus	No Estrus	*SEM*	*p*-Value (*t*-Test)	*p*-Value (Kruskal–Wallis)
Faith’s Phylogenetic Diversity	9.45	8.73	0.42	0.189	0.344
Observed Features	39.57	33.13	3.04	0.151	0.131
Shannon Index	4.17	3.36	0.25	0.048	0.098
Pielou’s Evenness	0.80	0.67	0.03	0.034	0.144

^1^ Uterine microbiome samples were collected on day 15 of the estrous cycle from the uterine horns ipsilateral and contralateral to the corpus luteum for entire (V1–V9 hypervariable regions) 16s rRNA gene sequencing. Non-parametric test (Kruskal–Wallis) is also shown because some data failed to meet the assumptions of normality. Estrus was evaluated on experimental day 0 using an estrus detection aid and cows were considered to have expressed estrus when ≥50% of the rub off coating was removed from the estrus detection patch. SEM: Standard error of the mean.

**Table 3:** Differences in mean relative abundance at the genus level between ipsilateral and contralateral uterine horns in non-pregnant cows on day 15 of the estrous cycle ^1^.

Genus	Ipsilateral (%)	Contralateral (%)	*SEM*	*p*-Value
*Butyrivibrio*	0.14	1.53	0.22	0.003
*Bacteroidales RF16 group*	0.37	0.00	0.08	0.013
*Cutibacterium*	3.31	8.11	1.41	0.025
*Clostridia UCG-014*	0.42	0.04	0.09	0.025
*BD7-11*	0.07	0.32	0.07	0.033
*Bacteroidales BS11 gut group*	0.12	0.43	0.10	0.037
*Ruminococcus*	0.40	1.03	0.21	0.045

^1^ Uterine microbiome samples were collected on day 15 of the estrous cycle from the uterine horns ipsilateral and contralateral to the corpus luteum for entire (V1–V9 hypervariable regions) 16s rRNA gene sequencing. Non-parametric test (Kruskal–Wallis) was performed because some data failed to meet the assumptions of normality. SEM: Standard error of the mean.

**Table 4:** Differences in mean relative abundance at genus level between ipsilateral and contralateral uterine horn using paired data (paired by cow) ^1^.

Genus	Ipsilateral (%)	Contralateral (%)	*SEM*	*p*-Value (Paired *t*-Test)	*p*-Value (Wilcoxon)
*Butyrivibrio*	0.06	1.46	0.25	0.004	0.004
*Bacteroidales RF16 group*	0.34	0.00	0.07	0.028	0.017
*Bacillus*	0.00	0.64	0.14	0.030	0.017
*Clostridia UCG-014*	0.48	0.00	0.11	0.031	0.008
*Cutibacterium*	2.60	8.77	1.62	0.043	0.018
*Veillonellaceae UCG-001*	0.25	0.00	0.06	0.043	0.035
*Bacteriodales BS11 gut group*	0.00	0.40	0.10	0.045	0.035
*Ruminococcus*	0.24	0.77	0.18	0.016	0.088

^1^ Uterine microbiome samples were collected on day 15 of the estrous cycle from the uterine horns ipsilateral and contralateral to the corpus luteum for entire (V1–V9 hypervariable regions) 16s rRNA gene sequencing. Non-parametric test (Wilcoxon) is also shown because some data failed to meet the assumptions of normality. SEM: Standard error of the mean.

**Table 5:** Differences in mean relative abundance at the genus level between cows that expressed estrus and cows that failed to express estrus ^1^.

Genus	Estrus (%)	No Estrus (%)	*SEM*	*p*-Value
*Rikenellaceae RC9 gut group*	4.32	1.59	0.52	0.002
*Bacteroidales UCG-001*	1.33	0.06	0.32	0.014
*Lachnospiraceae AC2044 group*	0.98	0.17	0.18	0.016
*Vibrionimonas*	0.94	2.24	0.34	0.023
*Burkholderia-Caballeronia-Paraburkholderia*	0.50	0.04	0.11	0.027
Family *Chitinophagaceae*	0.03	0.16	0.03	0.028
*dgA-11 gut group*	0.00	0.06	0.02	0.035
*Pseudobutyrivibrio*	0.34	0.00	0.09	0.045

^1^ Uterine microbiome samples were collected on day 15 of the estrous cycle from the uterine horns ipsilateral and contralateral to the corpus luteum for entire (V1–V9 hypervariable regions) 16s rRNA gene sequencing. Estrus was evaluated on experimental day 0 using an estrus detection aid and cows were considered to have expressed estrus when ≥50% of the rub off coating was removed from the estrus detection patch. Non-parametric test (Kruskal–Wallis) was performed because some data failed to meet the assumptions of normality. SEM: Standard error of the mean.

## Data Availability

Data are available upon request.
